# Case Report: Preoperative ultrasonographic diagnosis of accessory cavitated uterine malformation: a case series report and narrative review

**DOI:** 10.3389/fonc.2025.1658448

**Published:** 2025-10-03

**Authors:** Tingting Shen, Hongxia Yuan, Hong Cao, Junhong Liu, Xiangling Duan, Shasha Chen, Rong Tian, Xingxing Duan

**Affiliations:** Department of Ultrasonic Medicine, Changsha Maternal and Child Health Hospital, Changsha, China

**Keywords:** ACUM, accessory cavitated uterine malformation, Müllerian duct anomaly, uterine diseases, dysmenorrhea

## Abstract

**Objectives:**

Accessory cavitated uterine malformation/mass (ACUM) is an extremely rare uterine malformation that is frequently misdiagnosed preoperatively. This study presents three ACUM cases accurately diagnosed by preoperative ultrasonography in our hospital. Through a comprehensive literature review, we systematically summarize its characteristic sonographic findings and key points for differential diagnosis, aiming to enhance sonographers’ recognition of ACUM and improve the accuracy of preoperative diagnoses.

**Materials and methods:**

We collected three ACUM cases diagnosed in our hospital from January 2023 to April 2025. The general clinical information, ultrasound and radiological findings, pathological reports, and surgical records were retrospectively analyzed. A total of 13 previous literature reports, including a total of 39 ACUM cases, were also reviewed.

**Results:**

The comprehensive analysis of 39 previously reported ACUM cases and three confirmed cases from our institution revealed the following:

1. The mean age of the ACUM patients was 25.9 ± 6.5 years.

2. The primary clinical presentations of ACUM consisted of dysmenorrhea (83.3%) and lower abdominal pain (47.6%), with additional findings of dyspareunia (9.5%), difficult defecation (2.4%), and primary infertility (2.4%).

3. The ultrasonographic features are as follows:

a) ACUM typically appeared as a thick-walled cystic mass not connected to the uterine cavity.

b) The mean maximum outer diameter was 34.3 ± 11.7 mm (range 16–64 mm), with a median of 31.5 mm.

c) The cyst was often surrounded by a homogeneous thick muscular layer (83.3%) and exhibited ring-like or semi-ring-like vascular signals (19.0%).

d) The cystic cavity typically exhibited a ground-glass appearance (64.3%).

e) Only seven cases (16.7%) displayed clearly identifiable endometrial lining on ultrasound examination.

4. Details of diagnostic accuracy are as follows:

a) The preoperative ultrasound diagnostic concordance rate was 47.6%.

b) ACUM was most frequently misdiagnosed as uterine leiomyoma (28.6%) and cystic adenomyosis (21.4%), with one case (2.4%) misdiagnosed as type II rudimentary horn uterus.

**Conclusion:**

ACUM is an exceedingly rare lesion that is particularly prone to misdiagnosis. ACUM should be considered in young female patients with severe dysmenorrhea and imaging findings of a normal uterine cavity and bilateral ovaries and a thick-walled cystic mass within the myometrium that does not communicate with the uterine cavity. Familiarity with the ultrasound manifestations of ACUM can help sonographers make timely and accurate diagnoses, assisting clinicians in choosing appropriate treatment methods and alleviating patient suffering.

## 1 Introduction

Accessory cavitated uterine malformation/mass (ACUM) is a rare obstructive uterine developmental anomaly characterized by a non-communicating cystic lesion within the myometrium on the lateral side of the uterus below the round ligament attachment (1). Due to limited research, population-based epidemiological data are lacking, and most studies consist of case reports. Patients with ACUM often experience severe dysmenorrhea and lower abdominal pain, which respond poorly to analgesic therapy (2). Owing to the insufficient clinical awareness of ACUM, it has been frequently misdiagnosed as cystic adenomyosis, uterine leiomyoma, or rudimentary horn uterus. These diagnostic errors often lead to delayed treatment and significantly compromise the patient’s quality of life (2–6). Ultrasonography, with non-invasive, high-resolution, and real-time dynamic imaging capabilities, is the preferred modality for gynecological diagnosis. High-frequency transvaginal ultrasound combined with four-dimensional gynecological ultrasound reconstruction technology enables the precise evaluation of lesion morphology, spatial location, and anatomical relationship with the uterine cavity. However, ultrasound imaging data on ACUM are limited. This study characterizes the sonographic findings in three ACUM cases, compares them with existing publications, and synthesizes critical diagnostic and differential diagnostic features, with the objectives of improving clinicians’ recognition of ACUM and enhancing preoperative diagnostic accuracy.

## 2 Materials and methods

This study was conducted according to the principles outlined in the Declaration of Helsinki and was approved by the Clinical Research Ethics Committee of Changsha Maternal and Child Health Care Hospital. Written informed consent was obtained from all participants.

We collected three ACUM cases confirmed by gynecological surgery and pathological diagnosis in our hospital between January 2023 and April 2025. All three ACUM patients underwent both ultrasound and magnetic resonance imaging (MRI) examinations preoperatively, and each underwent laparoscopic uterine mass resection, with both surgical and pathological diagnoses confirming ACUM.

This study is a narrative review conducted in accordance with the Scale for the Assessment of Narrative Review Articles (7) guidelines to enhance methodological transparency and reporting quality. Literature retrieval was performed using the PubMed and Web of Science databases, covering publications from the inception of each database to July 29, 2023. The search terms applied were “ACUM,” “accessory cavitated uterine malformation,” or “accessory cavitated uterine mass.” The initial search yielded 30 articles. After stepwise screening, 13 studies meeting the eligibility criteria were included, comprising a total of 39 surgically and pathologically confirmed ACUM cases. These were combined with three confirmed cases from our institution for comprehensive analysis. The inclusion criteria were (1) surgically and pathologically confirmed ACUM, (2) the availability of detailed ultrasonographic examination data, and (3) English full-text articles. The exclusion criteria comprised the absence of ultrasound examination, insufficient imaging data, the lack of pathological confirmation, and the unavailability of full-text articles.

## 3 Results

### 3.1 Case reports

#### 3.1.1 Case 1

**Figure 1:**
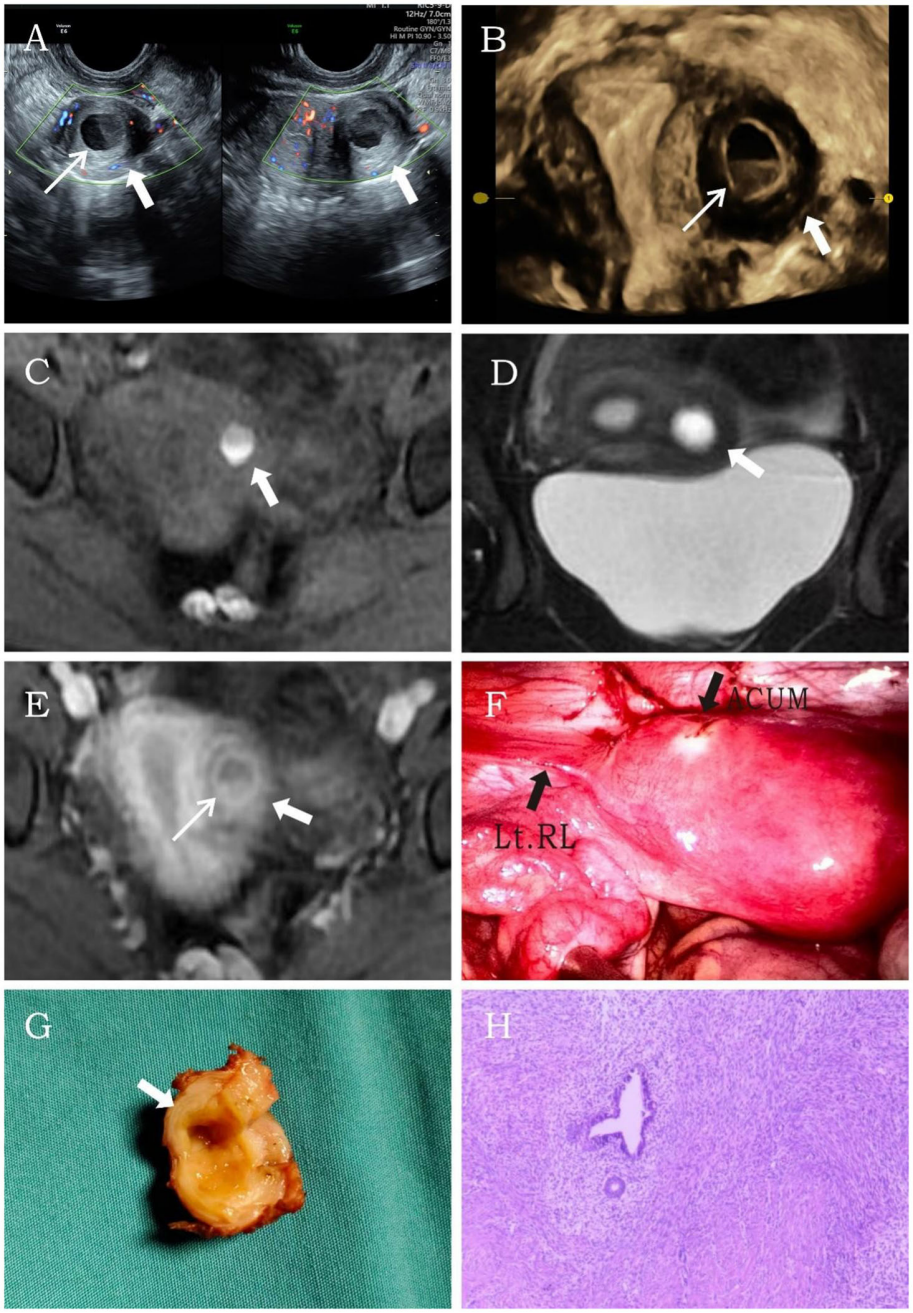
Representative image of clinical data for the first case. **(A, B)** Transvaginal and four-dimensional ultrasound images reveal a thick-walled cystic mass (thick white arrow) within the left lateral wall of the uterine myometrium. A thin endometrial-like ring (thin white arrow) is seen along the inner wall of the cavity, while the remaining uterine structure appears normal. **(C–E)** MRI demonstrates an abnormal signal focus (thick white arrow) in the left lateral myometrium. The T1-weighted fat-suppressed sequence shows high signal intensity within the cavity resembling hematometra. Post-contrast imaging reveals a thin endometrial-like slightly hyperintense signal (thin white arrow) along the inner wall. The T2-weighted image displays a low signal surrounding the inner wall, similar to the myometrium. **(F)** Laparoscopy shows a protruding mass at the insertion site of the round ligament of the uterus. **(G)** After incision, the cystic cavity is surrounded by a regular, thick layer of muscular tissue (thick white arrow). **(H)** Histopathological image (H&E, ×10). The submitted smooth muscle tissue is partially lined by hyperplastic endometrium, with scattered endometrial glands and stroma within the muscular wall.

A 14.5-year-old female patient, unmarried and nulliparous, was admitted with dysmenorrhea for 1 year. Menarche occurred at 13 years, with dysmenorrhea developing 6 months later, progressively worsening throughout the menstrual cycle, and even starting 2 to 3 days before menstruation. She had been diagnosed with cystic adenomyoma by ultrasound examination at a local hospital 1 year earlier. Physical examination identified a mass in the left uterine wall with moderate consistency, limited mobility, and no tenderness. Laboratory tests showed no abnormalities. Transvaginal sonography conducted at our institution identified a well-circumscribed, thick-walled cystic mass (24 × 20 × 22 mm) in the left uterine myometrium, featuring a ground-glass echogenic cavity (14 × 11 × 12 mm) with characteristic endometrial lining (1.0 mm) and a surrounding hypoechoic muscular rim (4.6 mm). The uterine cavity appeared normal, and no abnormalities were detected in either adnexal region. Color Doppler flow imaging (CDFI) revealed a semicircular blood flow signal surrounding the cystic mass (**Figures 1A, B**). The ultrasound diagnosis was ACUM. MRI showed a round abnormal signal focus in the left uterine myometrium with heterogeneous internal signals: slightly hyperintense (upper layer) and hyperintense (lower layer) on T1WI (**Figure 1C**) and hyperintense on T2WI (**Figure 1D**). Contrast-enhanced imaging showed gradual mild-to-moderate enhancement of the surrounding cystic wall (**Figure 1E**). The MRI diagnosis was cystic adenomyosis or rudimentary horn uterus. The patient was diagnosed with ACUM by laparoscopic surgery (**Figures 1F–H**) and underwent ACUM resection. During the 12-month postoperative follow-up, the patient remained asymptomatic with no recurrence.

#### 3.1.2 Case 2

**Figure 2:**
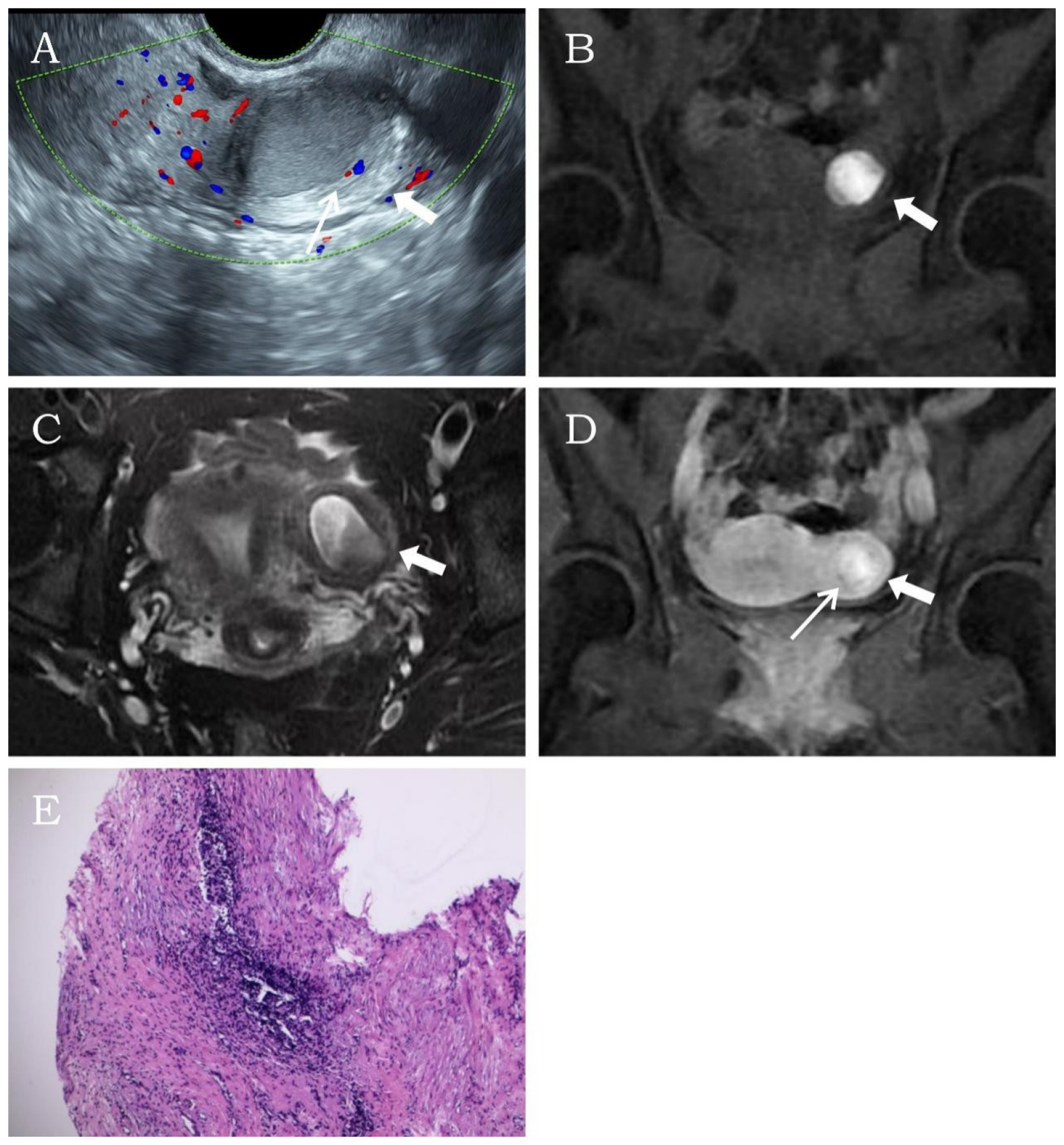
Representative image of clinical data for the second case. **(A)** Transvaginal ultrasound reveals a thick-walled cystic mass (thick white arrow) within the left lateral wall of the uterine myometrium. A thin endometrial-like ring (thin white arrow) is observed along the inner wall of the cavity, while the remaining uterine structure appears normal. **(B**–**D)** MRI demonstrates an abnormal signal focus (thick white arrow) in the left lateral myometrium, exhibiting heterogeneous internal signals. Post-contrast imaging shows a thin endometrial-like slightly hyperintense signal (thin white arrow) along the inner wall. The T2-weighted image displays a low signal surrounding the inner wall, similar to the myometrium. **(E)** Histopathological image (H&E, ×10). The submitted smooth muscle tissue is partially lined by endometrium, with scattered endometrial glands and stroma within the muscular wall.

A 26-year-old married nulliparous woman was admitted with a 2-year history of lower abdominal pain. The patient had developed lower abdominal pain 2 years earlier without obvious triggers. The pain occurred either at the end of menstruation or during menstrual onset, lasting variably from 2 to 7 days. It presented as intermittent colicky pain without progressive worsening or other discomfort. Physical examination revealed a moderately firm, minimally mobile, non-tender mass in the left uterine wall. Laboratory tests showed no abnormalities. Transvaginal sonography revealed a well-circumscribed, thick-walled cystic mass (37 × 28 × 34 mm) with regular morphology in the left uterine myometrium, featuring a ground-glass echogenic cavity (15 × 18 × 22 mm) with characteristic endometrial lining (1.2 mm) and a surrounding hypoechoic muscular rim (5.6 mm). The cystic mass showed no communication with the endometrial cavity. No significant abnormalities were detected in the uterine cavity or bilateral adnexal regions. CDFI revealed a circular blood flow signal surrounding the cystic mass (**Figure 2A**). This finding resulted in a diagnosis of ACUM. The MRI examination demonstrated a round, heterogeneously signal-intense lesion in the left uterine wall, showing high signal intensity on T1WI (**Figure 2B**) and mixed high-low signal intensity on T2WI (**Figure 2C**), contrast-enhanced imaging showed no significant central enhancement (**Figure 2D**), leading to a diagnostic consideration of cystic adenomyosis or rudimentary horn uterus. The patient was diagnosed with ACUM by laparoscopic surgery (**Figure 2E**) and underwent complete ACUM excision. The patient has remained asymptomatic without recurrence during 24 months of postoperative follow-up.

#### 3.1.3 Case 3

**Figure 3:**
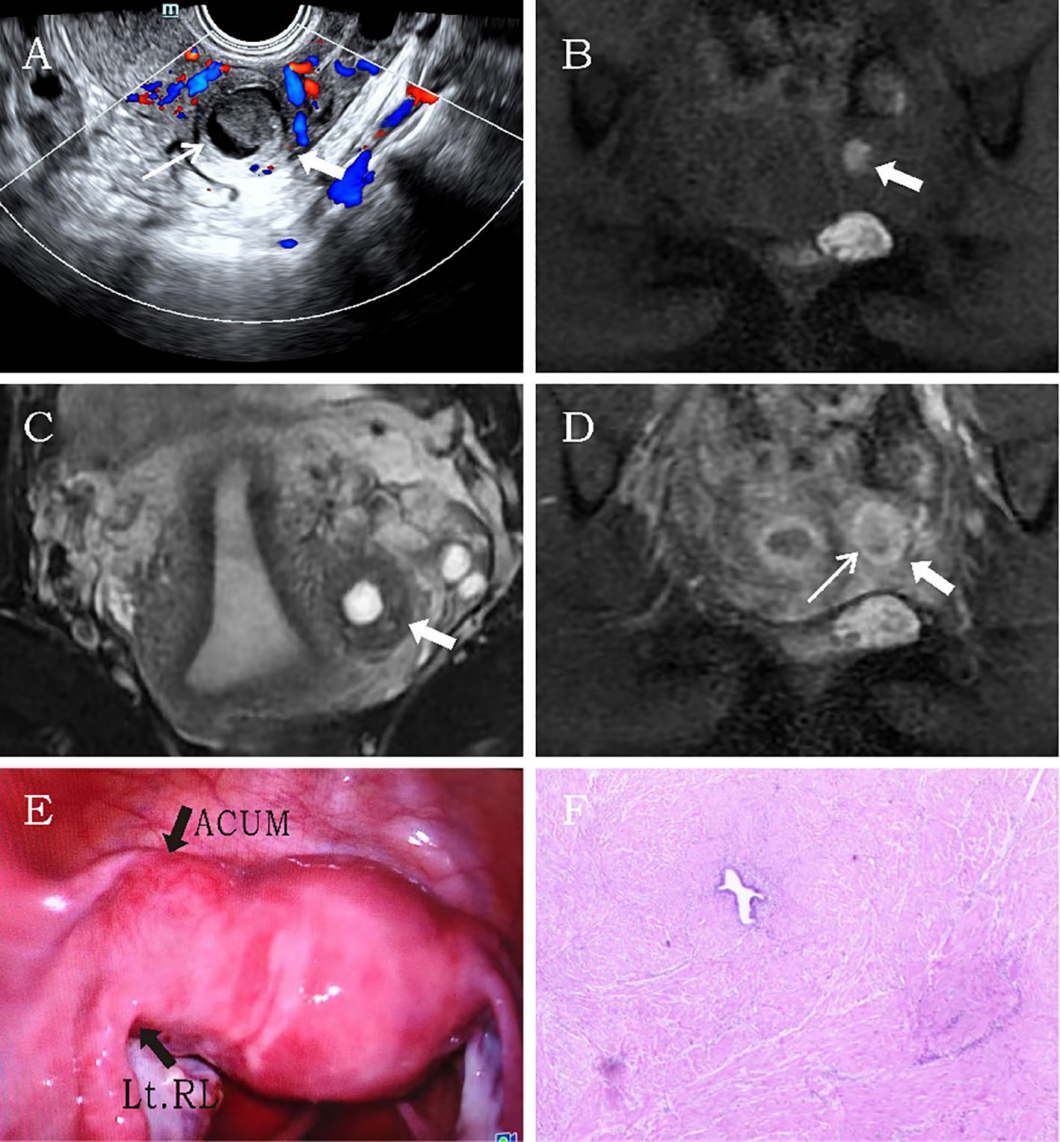
Representative image of clinical data for the third case. **(A)** Transvaginal ultrasound demonstrates a thick-walled cystic mass (thick white arrow) within the left lateral myometrium, showing a thin endometrial-like lining (thin white arrow) along the cavity wall. The remaining uterine architecture appears unremarkable. **(B–D)** MRI reveals an abnormal signal focus (thick white arrow) in the left myometrial wall. The lesion exhibits central hyperintensity on both T1- and T2-weighted images. Post-contrast imaging displays thin rim-like enhancement (thin white arrow) along the cavity wall, while T2-weighted imaging shows a hypointense rim surrounding the cavity, consistent with myometrial signal characteristics. **(E)** Laparoscopic visualization identifies a protruding mass at the uterine round ligament insertion site. **(F)** Histopathological examination (H&E, ×10). The specimen consists of smooth muscle tissue partially lined by hyperplastic endometrium, with interspersed endometrial glands and stroma within the muscular wall.

A 34-year-old married woman (gravida 2 para 2) was admitted due to intermittent lower abdominal pain for over 1 month. The patient had a history of mild dysmenorrhea. She experienced severe pain in the left lower abdomen 1 month earlier. She was diagnosed with acute pelvic inflammatory disease by external ultrasound examination, which revealed a heterogeneous echogenic mass within the left uterine myometrium. The pain resolved after anti-inflammatory and antispasmodic treatments. However, the patient experienced a recurrence of left lower abdominal pain 1 day ago, characterized by persistent colicky pain that was unresponsive to medication therapy. The patient had no contributory medical or family history. Physical examination revealed mild protrusion of the left anterior uterine wall without tenderness. All laboratory findings were within the normal limits. Ultrasonography demonstrated a well-circumscribed, regularly shaped thick-walled cystic mass (29 × 30 × 31 mm) in the left uterine wall. The lesion contained a cystic cavity (17 × 14 × 18 mm) with ground-glass echogenicity and a solid medium-echo component (13 × 12 × 15 mm). The cystic lumen was lined by a 1.3-mm-thick circumferential endometrial layer and surrounded by a 5.1-mm-thick hypoechoic myometrial rim. The uterine cavity appeared normal with unremarkable bilateral ovaries. CDFI revealed a circular blood flow signal surrounding the cystic mass (**Figure 3A**). The imaging characteristics confirmed a diagnosis of ACUM. MRI revealed a round abnormal signal intensity lesion within the left broad ligament of the uterine myometrium. The lesion demonstrated central hyperintensity with slightly hypointense margins containing scattered punctate hyperintense foci on both T1WI and T2WI (**Figures 3B, C**). Contrast-enhanced imaging showed no significant central enhancement (**Figure 3D**). These MRI features were suggestive of ACUM. The patient was diagnosed with ACUM by laparoscopic surgery (**Figures 3E, F**) and underwent ACUM excision. The patient remained asymptomatic with no recurrence during the 6-month postoperative follow-up period.

### 3.2 Literature review

**Table 1** summarizes the clinical and ultrasonographic characteristics of 39 ACUM cases reported in previous publications, along with three additional cases from our institution. The results revealed are detailed below.

**Table 1:** General characteristics and detailed ultrasound findings of 42 ACUM patients.

Reference	Number of cases	Age	GPA	Main symptom	US diagnosis	Location	Cyst size (mm)	Endometrial lining (Y/N)	Thick muscular wall (Y/N)	Internal echo	Circular blood flow (Y/N)	Endometriosis (Y/N)
Ruijie Sun (8)	9	30	G1P1	Progressive dysmenorrhea	ACUM	R	28	NA	Y	Ground-glass echogenicity	Y	N
		25	G0	Progressive dysmenorrhea	ACUM	R	28	NA	Y	Ground-glass echogenicity	Y	N
		30	G0	Progressive dysmenorrhea	ACUM	L	23	NA	Y	Ground-glass echogenicity	Y	N
		20	G0	Progressive dysmenorrhea	ACUM	R	24	NA	Y	Ground-glass echogenicity	Y	N
		39	G0	Progressive dysmenorrhea	ACUM	R	28.3	NA	Y	Strongly echogenic	Y	N
		32	G5P3	Progressive dysmenorrhea	ACUM	L	26.3	NA	Y	Ground-glass echogenicity	Y	N
		31	G2P2	Progressive dysmenorrhea	ACUM	L	16.3	NA	Y	Ground-glass echogenicity	Y	N
		31	G1P1	Mild dysmenorrhea	ACUM	L	22	NA	Y	Ground-glass echogenicity	Y	N
		18	G0	Progressive dysmenorrhea	ACUM	R	23.3	NA	Y	Ground-glass echogenicity	Y	N
Sanjay MhalaSakant khaladkaR (9)	1	19	G0	dysmenorrhea	ACUM	R	29	Y	Y	Anechoic	NA	Y
Qicai Hu (10)	3	30	G1P1	Progressive dysmenorrhea	Uterine leiomyoma	R	31	NA	Y	Ground-glass echogenicity	Y	N
		25	G0	dysmenorrhea	Cystic adenomyosis	R	30	NA	Y	Anechoic	NA	N
		30	G0	Lower abdominal pain;Progressive dysmenorrhea	Cystic adenomyosis	L	34	NA	Y	Ground-glass echogenicity	NA	N
Fenglian Deng (11)	1	30	G0	Lower abdominal pain Progressive dysmenorrhea	Cystic adenomyosis	R	64	NA	Y	Ground-glass echogenicity with embedded hyperechoic masses	Y	N
Dan Boitor-Borza (12)	1	35	G2P2	Progressive dysmenorrhea Dyspareunia	ACUM	L	64	NA	Y	Ground-glass echogenicity interspersed with anechoic areas	Y	N
Michael Strug (13)	1	31	G0	Lower abdominal pain Progressive dysmenorrhea Primary infertility	ACUM	L	20	NA	Y	Ground-glass echogenicity	Y	Y
Rana Mondal (14)	8	19	G0	Progressive dysmenorrhea	Uterine leiomyoma	R	40	NA	NA	NA	NA	N
		20	G0	Lower abdominal pain	Cystic adenomyosis	L	55	NA	Y	Ground-glass echogenicity	NA	N
		19	G0	Lower abdominal pain Progressive dysmenorrhea	Uterine leiomyoma	L	43	NA	Y	Ground-glass echogenicity	NA	N
		34	G0	Lower abdominal pain	Uterine leiomyoma	R	53	Y	Y	Ground-glass to hypoechoic echogenicity	NA	N
		15	G0	Progressive dysmenorrhea	Cystic adenomyosis	L	39	NA	NA	Hyperechoic masses within ground-glass echogenicity	NA	N
		27	G0	Lower abdominal pain Progressive dysmenorrhea Dyspareunia Dyschezia	Uterine leiomyoma	R	40	NA	NA	Hyperechoic	Y	N
		29	G0	Dyspareunia	Cystic adenomyosis	L	54	NA	NA	NA	NA	NA
		22	G0	Lower abdominal pain	Cystic adenomyosis	R	30	NA	Y	Ground-glass echogenicity	NA	N
Weronika Zajaczkowska (1)	1	24.6	NA	Lower abdominal pain Progressive dysmenorrhea	Cystic adenomyosis	R	16	NA	Y	Isoechoic masses within anechoic areas	NA	N
Japleen Kaur (15)	1	32	G2P2	Progressive dysmenorrhea Lower abdominal pain Dyspareunia	ACUM	L	29	NA	Y	Ground-glass echogenicity	NA	N
Mohit Veerkumar Shah (16)	1	28	G0	Progressive dysmenorrhea Lower abdominal pain	ACUM	R	32	Y	Y	Ground-glass echogenicity	Y	N
Tharani Putta (17)	6	17	G0	Progressive dysmenorrhea Lower abdominal pain	Uterine leiomyoma	R	30	NA	Y	Ground-glass echogenicity	NA	N
		23	G0	Progressive dysmenorrhea Lower abdominal pain	Uterine leiomyoma	L	33	NA	Y	Hyperechoic masses within an anechoic area	NA	Y
		21	G0	Lower abdominal pain	Uterine leiomyoma	L	40	NA	Y	Ground-glass echogenicity	NA	N
		32	G2P2	Dysmenorrhea	Uterine leiomyoma	L	43	NA	Y	Ground-glass echogenicity	NA	N
		26	G0	Dysmenorrhea	Uterine leiomyoma	R	33	NA	Y	NA	NA	N
		19	G0	Dysmenorrhea	Uterine leiomyoma	L	42	NA	Y	Ground-glass echogenicity	NA	N
Sevellaraja Supermaniam (18)	2	22	G0	Progressive dysmenorrhea	Cystic adenomyosis	R	36	NA	Y	Ground-glass echogenicity	NA	N
		36	G0	Dysmenorrhea	ACUM	R	33	Y	Y	Ground-glass echogenicity	NA	Y
P Acién (2)	4	36	G2P2	Lower abdominal pain	ACUM	L	50	NA	Y	Ground-glass echogenicity	NA	N
		20	G0	Lower abdominal pain Dysmenorrhea	Type II rudimentary horn uterus	L	40	NA	NA	Ground-glass echogenicity	NA	N
		18	G0	Lower abdominal pain Dysmenorrhea	ACUM	L	26	NA	NA	Ground-glass echogenicity	NA	N
		19	G0	Lower abdominal pain Dysmenorrhea	Uterine leiomyoma	L	20	NA	NA	NA	NA	N
Our casese	Case 1	14.5	G0	Dysmenorrhea	ACUM	L	24	Y	Y	Ground-glass echogenicity	Y	N
	Case 2	26	G0	Lower abdominal pain	ACUM	L	37	Y	Y	Ground-glass echogenicity	Y	N
	Case 3	34	G2P2	Lower abdominal pain	ACUM	L	31	Y	Y	Isoechoic masses within ground-glass echogenicity	Y	N

The mean age of the ACUM patients was 25.9 ± 6.5 years (range: 14.5–39). Dysmenorrhea (83.3%) and lower abdominal pain (47.6%) were the most common clinical manifestations, while a minority of patients presented with dyspareunia (9.5%), difficult defecation (2.4%), or primary infertility (2.4%). ACUM lesions predominantly occurred in the left uterine wall (54.8%), with the remainder (45.2%) developing in the right wall.

Ultrasonography demonstrated ACUM as a thick-walled cystic lesion (mean maximal diameter 34.3 ± 11.7 mm; range: 16–64 mm; median 31.5 mm) without uterine cavity communication. The key characteristics included a surrounding homogeneous myometrial layer (83.3%), ground-glass internal echogenicity (64.3%), and variable echo patterns (anechoic 4.8%, hyperechoic 2.4%, strongly echogenic 2.4%, other 30.4%). The endometrial lining was identifiable in 16.7% of the cases, with 42.9% demonstrating characteristic peripheral circular/semicircular vascularity on Doppler imaging.

Among these 42 cases, the preoperative ultrasound diagnosis demonstrated a 45% concordance rate with surgical findings. The most frequent misdiagnoses included uterine leiomyoma (28.6%) and cystic adenomyosis (21.4%), with one case (2.4%) misinterpreted as type II rudimentary horn uterus.

## 4 Discussion

ACUM is an exceptionally rare obstructive uterine developmental anomaly first described by O. Live in 1912 (14). It is not included in the Müllerian duct anomaly classification systems of the European Society of Human Reproduction and Embryology/European Society for Gynaecological Endoscopy or the American Society for Reproductive Medicine (19–21). The Sino-European Consensus on ACUM, published in June 2025, formally classified ACUM as a distinct clinical entity for the first time. This landmark consensus underscores that ACUM has long been an underrecognized and underdiagnosed condition in clinical practice (22). ACUM was historically reported under various designations, including juvenile cystic adenomyoma (JCA) (2–5) and uterus-like mass (6), until its formal nomenclature was first established in 2021 by Acién, a European reproductive tract specialist (2). However, the exact incidence of ACUM remains undetermined. To date, a total of 31 case reports have been published, documenting 125 collective ACUM cases. The largest case series was reported by Naftalin et al. (23).

The pathogenesis of ACUM remains incompletely understood, although most researchers classify it as a distinct form of Müllerian duct anomaly. Current evidence suggests that it may arise from ectopic or duplicated Müllerian tissue, potentially involving the dysfunction of the gubernaculum (which later develops into the round ligament) (24–26). Pathologically, ACUM is defined by three hallmark features: (1) a central cavity lined by functional endometrium capable of cyclic shedding, (2) the intraluminal accumulation of chocolate-colored hemolyzed blood products indicative of chronic hemorrhaging, and (3) concentric layers of regularly arranged smooth muscle fibers, forming a well-demarcated peripheral ring (27, 28).

ACUM predominantly occurs in nulliparous women under 30 years of age, although it can also affect women over 30 and multiparous women. In our study, the age distribution (14 patients >30 years and 28 patients ≤30 years) was consistent with previous reports (13, 29, 30), further validating this epidemiological pattern. Clinically, the patients predominantly present with severe dysmenorrhea (35/42, 83.3%) and lower abdominal pain (20/42, 47.6%). The pain frequently localizes to either the ipsilateral abdomen or the entire lower abdominal region, with marked exacerbation during menstruation or premenstrual onset. Pharmacological interventions often demonstrate limited efficacy (2, 10, 31). A proportion of patients may additionally present with dyspareunia (4/42, 9.5%) and dyschezia (1/42, 2.4%), consistent with previous descriptions of ACUM symptomatology (13, 29). A minority of patients remain asymptomatic and are incidentally diagnosed through laparoscopic examination or ultrasonography (14).

ACUM typically presents as a solitary lesion, although bilateral or multiple ACUMs may occur. Sun (8) documented a rare case with two ipsilateral ACUM masses in a single uterus. These lesions demonstrate considerable size variability (predominantly 2–4 cm in diameter), correlating with intracavitary hemorrhage volume. The largest reported ACUM measured 11 × 11 × 8 cm (32). The mean maximum outer diameter of the cystic masses in this study was 34.3 ± 11.7 mm (range: 16–64 mm), which was consistent with previous reports by Timmerman et al. (29) (31.5 ^mm^) and Dekkiche et al. (30) (33.5 mm).

The definitive diagnosis of ACUM relies on surgical exploration and pathological confirmation, with four essential criteria, namely:

1. An isolated accessory cavitated mass located at the uterine round ligament insertion site.
2. Normally developed uterine cavity, fallopian tubes, and ovaries.
3. Histopathological confirmation of endometrial lining in the accessory cavity with chocolate-colored fluid.
4. The absence of adenomyosis (although small foci may exist in the adjacent myometrium) (22, 28, 33).

Ultrasonography and MRI serve as the primary imaging modalities for ACUM evaluation, with transvaginal/transrectal intracavitary ultrasound being the first-line diagnostic approach (22, 23). Gynecological four-dimensional ultrasound imaging can directly demonstrate that ACUM does not communicate with the normally triangular uterine cavity. This provides reliable evidence for the anatomical localization of ACUM and its differential diagnosis from other obstructive uterine malformations (34). The characteristic sonographic appearance is a cystic mass located within the myometrium lateral to the uterine cornu. The cystic cavity often exhibits a ground-glass appearance, resembling the internal echoes of an endometriotic cyst, and occasionally exhibits moderate-to-high echogenicity (23, 29). In this study, 27 cases (64.3%) exhibited this characteristic, which is consistent with prior studies (29). Transvaginal ultrasonography can dynamically demonstrate characteristic cyclic changes in the endometrial lining corresponding to the menstrual phase, which serves as a pivotal diagnostic feature. During acute pain episodes, Doppler ultrasound typically reveals increased peripheral vascularity around the cyst, along with hypoechoic intracavitary contents. However, in our study, only a minority (16.7%) of the ACUM patients demonstrated clearly visible, typical endometrial lining structures within the cystic cavity. The diagnostic accuracy was only 47.6% when comparing preoperative ultrasound findings with postoperative pathological results in this study. This discrepancy primarily stems from an insufficient understanding of ACUM among previous researchers and misjudgments caused by cognitive bias. However, a study by Sun (8) and three cases from our institution demonstrated 100% preoperative diagnostic accuracy by ultrasound, indicating that ACUM can be reliably diagnosed by identifying its characteristic sonographic features.

ACUM appears on MRI as a solitary, round intramyometrial mass. On T2WI, the cystic cavity may demonstrate a thin, slightly hyperintense lining resembling the endometrium, with mild post-contrast enhancement. A hypointense rim on T2WI typically surrounds the cavity. T1WI reveals hyperintense intracavitary contents, indicative of hemorrhagic components (14, 27, 34). Unlike ultrasound, MRI is operator-independent with excellent reproducibility, making it particularly suitable for patients with obesity, bowel gas interference, or complex pelvic anatomy.

Hysteroscopy serves as a valuable adjunct diagnostic tool for ACUM. Previous studies have emphasized that ACUM diagnosis requires both the demonstration of normal uterine cavity morphology and the exclusion of other congenital uterine anomalies (35). Hysteroscopy provides dual diagnostic values for ACUM. First, it enables the direct visualization of the non-communication between ACUM and the endometrial cavity, which serves as a key diagnostic criterion to differentiate it from other Müllerian anomalies such as rudimentary horn or Robert uterus. Second, it allows for the precise evaluation of ACUM’s mechanical impacts on the uterine cavity, including cavity compression/deformation and endometrial abnormalities, which may potentially affect embryo implantation. These hysteroscopic findings are particularly valuable for future investigations into the potential association between ACUM and infertility (36–39).

ACUM requires differential diagnosis from uterine leiomyoma, cystic adenomyosis, type II rudimentary uterine horn, and Robert’s uterus.

Uterine leiomyoma: Patients with conventional uterine leiomyomas typically present without dysmenorrhea or lower abdominal pain, often with a documented history of uterine leiomyomas. These masses may develop in any location within the myometrium and frequently present as multiple lesions, characteristically demonstrating a whorled internal echo pattern on ultrasonography. Notably, ACUM is often misdiagnosed as the cystic degeneration of uterine leiomyoma. However, true cystic degeneration remains relatively uncommon, constituting only 4% of all leiomyoma degeneration cases (18), and seldom exhibits hemorrhagic content.

Cystic adenomyosis: Patients with this condition are typically older in age, and dysmenorrhea often occurs following cesarean section or curettage procedures. On ultrasonography, cystic adenomyosis may be seen in any location within the myometrium, usually presenting with ill-defined margins and lacking the characteristic hypoechoic circumferential rim. Additionally, other areas of the myometrium often exhibit features of adenomyosis, and the endometrial–myometrial junctional zone is frequently disrupted.

Type II rudimentary uterine horn (with functional endometrium): This condition predominantly presents with adolescent dysmenorrhea and demonstrates characteristic sonographic findings of an asymmetric bicornuate uterus, featuring a fundal indentation >1 cm and comprising a unicornuate uterus on one side and a hypoplastic rudimentary horn on the contralateral side. The two components may be immediately adjacent or spatially separated, connected by muscular or fibrous bands. The rudimentary horn typically displays endometrial-like moderate hyperechogenicity or contains dense punctate hypoechoic foci while maintaining anatomical continuity with the ipsilateral fallopian tube. In contrast, ACUM exhibits normal fundal and endometrial cavity morphology with clearly visualized bilateral cornua, presenting as an intramyometrial lesion without communication to the ipsilateral fallopian tube.

Robert’s uterus: This anomaly typically presents with adolescent dysmenorrhea and demonstrates the characteristic sonographic features of an asymmetric septate uterus with normal fundal contour but lacks a functional endometrial cavity. The malformation consists of (1) a unicornuate hemi-uterus with cervical communication appearing as a tubular structure and (2) an obstructed hemi-cavity containing dense punctate hypoechoic foci (hematometra) that may communicate with the main cavity through a minute orifice, potentially accompanied by ipsilateral hydrosalpinx. In contrast, ACUM maintains a normal uterine cavity morphology with clearly visualized bilateral cornua, presenting as an intramyometrial lesion without communication to either the endometrial cavity or ipsilateral fallopian tube.

Misdiagnosis or missed diagnosis of ACUM may delay appropriate treatment, with progressively worsening dysmenorrhea, chronic pelvic pain, or dyspareunia significantly impairing the patient’s quality of life. An incorrect diagnosis of cystic degeneration of uterine leiomyoma or cystic adenomyosis will directly compromise clinical decision-making, leading to inappropriate management strategies.

Müllerian duct anomalies have a prevalence of up to 7% in the general population, with an incidence of up to 25% in women with infertility or recurrent pregnancy loss (39–41). In our study cohort of 42 ACUM patients, 10 (23.8%) had a childbirth history, while only one (2.4%) presented with primary infertility. This incidence is comparable to the rate reported by Strug et al. (13) (4.3%), suggesting a potential association between ACUM and primary fertility. However, the exact relationship and underlying physiological mechanisms remain to be clarified in larger-scale studies due to limited sample sizes.

Surgical management remains the mainstay treatment for ACUM, with the primary objectives of excising the accessory cavitated mass and relieving hematometra accumulation. Laparoscopic resection of the accessory cavity is the preferred approach and offers significant advantages including minimal invasiveness, reduced scarring, faster recovery, and decreased risk of pelvic adhesions compared with open surgery (14, 30). A study by Barrett-Chan et al. (42), reviewing data from 75 patients, showed that surgery improved the symptoms in 84% (*n* = 63/75) of cases. Similarly, all three patients in our institution experienced complete resolution of symptoms postoperatively, with no recurrence during follow-up. Ultrasound-guided absolute ethanol sclerotherapy of the accessory cavity represents an alternative low-risk intervention that provides significant symptomatic relief while avoiding surgical scarring and uterine rupture risks. However, it carries concerns regarding potential recurrence (23, 43). While pharmacological intervention is less commonly applied, a study by Knochenhauer et al. (44) suggested that norethindrone acetate suppression therapy may be a feasible option for managing severe pain and dysmenorrhea secondary to ACUM.

This study has several important limitations that warrant consideration. First, the relatively small sample size (*n* = 42) may compromise statistical power and the generalizability of the findings. Second, our search strategy exclusively utilized “ACUM” as the primary keyword, which potentially introduced selection bias by omitting clinically relevant cases reported under different diagnostic terminologies, such as JCA or uterus-like mass, that otherwise would meet the inclusion criteria. Third, the included cases were sourced from multiple medical institutions with variations in ultrasonographic diagnostic criteria (including equipment parameters and examination protocols), which may have compromised the comparability of the results. Fourth, the majority of studies lacked systematic postoperative follow-up, resulting in incomplete long-term outcome data, particularly the objective assessment of reproductive outcomes (e.g., pregnancy rates and live birth rates), which made it difficult to comprehensively evaluate the long-term impact of ACUM on fertility potential. These limitations collectively highlight the necessity for future investigations to (1) increase sample sizes to improve statistical validity, (2) refine the search strategies by including alternative diagnostic terminologies, (3) develop standardized diagnostic and therapeutic protocols, and (4) implement prospective follow-up studies with predefined clinical endpoints to obtain more robust evidence.

## 5 Conclusion

In conclusion, as a rare and frequently misdiagnosed disorder, ACUM should be considered in the differential diagnosis when young women present with severe dysmenorrhea or pelvic pain, particularly when imaging studies reveal (1) normal uterine cavity morphology, (2) bilaterally normal ovaries, and (3) a thick-walled cystic lesion within the myometrium. Proficiency in recognizing the characteristic sonographic features of ACUM enables early and accurate diagnosis by ultrasonographers, facilitating appropriate clinical management that is critical for improving the patient’s quality of life.

## Data Availability

The original contributions presented in the study are included in the article/supplementary material. Further inquiries can be directed to the corresponding author.
